# Strategies to overcome mental health stigma: Insights and recommendations from young people with major depressive disorder (MDD)

**DOI:** 10.1002/brb3.70028

**Published:** 2024-09-18

**Authors:** Katie Prizeman, Netta Weinstein, Ciara McCabe

**Affiliations:** ^1^ Department of Psychology and Clinical Language Sciences University of Reading Reading UK

**Keywords:** clinical depression, disclosure, loneliness, mental health, qualitative research, recommendations, social isolation, stigma, young people

## Abstract

**Background:**

Young people with depression are met with stigma related to their mental health, which exacerbates loneliness, social isolation, and depression symptoms. While disclosing depression could improve one's mental health, stigma can also make social interactions more challenging and reduce the likelihood of receiving treatment. This research explored young people's experiences with stigma and recommendations for addressing it.

**Methods:**

Semi‐structured interviews conducted with *N *= 35 young people aged 18–25 years (*M_age_* = 20.09) were analyzed with thematic analysis. Participants met the criteria for clinical depression using the Mood and Feelings Questionnaire (score >27) or had recently obtained a medical diagnosis (*N *= 18) of depression by a medical professional.

**Results:**

Participants faced stigma when deciding to disclose their depression, which fed into a vicious cycle influencing feelings of loneliness, social isolation, and withdrawal. Their recommendations for others to avoid this cycle can be summarized under three main themes: (1) Social affirmation: identify allies and build meaningful connections; (2) Self‐affirmation: build a constructive relationship with the self; and (3) Societal affirmation: structural changes are needed.

**Conclusions:**

The current research indicates that social, self‐, and societal affirmation are considered important for reducing the detrimental impacts of stigma. Policies and programs are needed that provide mental health support to young people, and public awareness campaigns that guide young people to appropriate resources (i.e., support and intervention) via governmental public health bodies.

## Graphical abstract

Stigma among depressed young people worsens loneliness, isolation, and symptoms, hindering social interactions and treatment. To break this cycle, 35 participant interviews recommended three forms of affirmation: social, self‐, and societal affirmation. Public awareness campaigns, policies, and programs supporting mental health are crucial to guiding young people to appropriate resources.

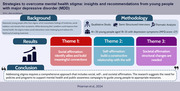

## 1 INTRODUCTION

Major depressive disorder (MDD) is a common mental illness that affects both adults and young people and impacts an estimated 3.8% of people worldwide (Thapar et al., 2012; World Health Organization, 2023). Prevalence rates in young people have risen sharply in the past decade, with those between the ages of 16 and 24 at the highest risk, with up to 5% prevalence by the end of adolescence (Australian Bureau of Statistics, 2007; Kessler et al., 2005; Thapar et al., 2022; WHO, 2021).

This is a serious cause for concern, as adolescence is a time of rapid social, emotional, and cognitive development, as well as important life changes (Malhi & Mann, 2018). Young people who experience depression symptoms may develop recurring depression, which can lead to the onset of other psychiatric disorders (i.e., bipolar disorder, anxiety, schizophrenia, etc.; Malhi & Mann, 2018), as well as eating disorders (Puccio et al., 2016) and functional somatic symptoms (Campo, 2012). In addition, depression can cause larger, long‐lasting deficits in interpersonal, educational, occupational, and social functioning (i.e., loneliness, social isolation, withdrawal, and judgment and discrimination because of one's mental illness). The social ramifications of depression, particularly the stigma associated with mental health, can intensify the illness's limitations for young people who experience it (Bailey, 1999; DeLuca, 2020; Weeks & Sullivan, 2019). Young people are particularly vulnerable to mental health stigmatization (Larson & Corrigan, 2008), making early intervention, depression prevention, and stigma reduction crucial among the younger population (Arboleda‐Flórez & Stuart, 2012; Javed et al., 2021; Pinfold et al., 2005; Stangl et al., 2019).

Mental health stigma is defined as circumstances, behavior, status, and/or identity that are linked to perceived inferiority (Price & Hollinsaid, 2022). It can take three forms. First, *public stigma* reflects negative attitudes, beliefs, and stereotypes that are directed toward people with mental illnesses by the public. Second, *internalized stigma* reflects the stigmatized person's own internalized negative beliefs, attitudes, and stereotypes that had been previously placed on them by society. Finally, *structural stigma* reflects laws, policies, and practices that result in unfair treatment of people with mental illnesses (Price & Hollinsaid, 2022). In all, the three forms of stigma pose a global barrier to help‐seeking behavior (Scott et al., 2015), engagement in care (Corrigan, 2004), and adherence to appropriate treatment (DiIorio et al., 2003; Link & Hatzenbuehler, 2016; Mahajan et al., 2008) among individuals suffering from depression.

All three forms of stigma can increase feelings of loneliness (a negative emotional state observed when there is a perceived gap between one's desired and actual social ties) and social isolation (the absence of social relationships where connections are limited or non‐existent) as a result of being left out of daily activities (Hinshaw, 2009; Matthews et al., 2016; Peplau & Perlman, 1982; Schulze & Angermeyer, 2003). These discriminatory and stigmatizing experiences have been described as being worse than the experience of depression itself (Vigo, 2016).

Furthermore, young people with depression must decide whether to disclose their stigmatized condition (Goffman & Goffman, 1963; Jones, 1984). This can be challenging, as they risk being stereotyped, rejected, discriminated against, misunderstood, and unsupported (Kelly & McKillop, 1996). This makes non‐disclosure an easier decision for some to avoid potentially awkward interactions, embarrassment, and negative judgments (Barney et al., 2006). Thus, stigma can prevent people from disclosing their depression and, ultimately, from getting the appropriate treatment and support (Garcia & Crocker, 2008). Research has also shown that contact between non‐stigmatized and stigmatized individuals can lessen stereotyping, prejudice, and stigmatization (Angermeyer & Corrigan, 2005). Therefore, disclosing depression can be beneficial for others as well as for the person living with a mental illness. Disclosure can assist people in receiving and providing social support to others who have stigmatizing attitudes and beliefs about mental illnesses. This may reduce feelings of loneliness and social isolation (Angermeyer et al., 2005).

Previous studies have suggested that social support, through the positive effects of social interactions, both directly and indirectly protects mental health from stressful and stigmatizing events (Acoba, 2024; Bjørlykhaug et al., 2022). Social support is understood as the emotional (e.g., encouraging someone), instrumental (e.g., assisting with housework), or informational (e.g., alerting someone to a job opportunity) support that one receives from others. This multidimensional concept is identified by the source of support (i.e., support from a family member, friend, and/or partner), each thought to have independent protective effects against depression (Gariépy et al., 2016; House et al., 1988; Thoits, 2011).

While limited research relating to mental health disclosure decisions exists (Brohan et al., 2012; McGrath et al., 2023), it is also unclear which sources or types of social support are most beneficial against depression and associated stigmas among the younger populations, where and how to find these sources, and their effect on feelings of loneliness, social isolation, and withdrawal. For instance, it is possible that in young adulthood, parental support is less valuable than it is in childhood. Furthermore, research on interventions incorporating advice from young people with lived depression stigma experiences is limited. This information is essential for the development of more focused social support programs, the targeting of younger populations in research, and the management of policymakers' recommendations to medical professionals on how to effectively combat stigmatizing practices and lessen the detrimental effects of stigma related to depression.

## 2 CURRENT STUDY

This study was developed to examine how young people (aged 16–25 years) who identify with MDD (have a clinical diagnosis or elevated depressive symptoms) experience stigma, their decisions to disclose their mental health, and what recommendations they have for others on how to strategically and intentionally identify supportive others to disclose one's depression and overcome stigma. The study pursued two research questions (RQs) to achieve this overarching goal:

RQ #1: What are the views on stigma among young people with depression? And how do they see stigma as related to feelings of loneliness, social isolation, withdrawal, and disclosing depression to others?

RQ #2: What recommendations do young people with depressive symptoms have for reaching out to others (i.e., challenges and warnings one would give other people about reaching out; is there a preferred mode of talking, such as online or in communities, etc., and tips to find these sources)? For instance, what strategies do young people with depression have for others to disclose their mental health and overcome stigma.

## 3 METHODS

### 3.1 Participants and recruitment

Young people (*N *= 35) between the ages of 16 and 25 (*M*_age_ = 20.09) who had high scores (>27) on the Mood and Feelings Questionnaire (MFQ; Costello & Angold, 1988) were recruited from the community utilizing advertisements and posters between December 1st, 2023, and January 31st, 2024. Participants were reimbursed for their time and effort by being entered into a draw for a £150 prize. After completing socio‐demographic questionnaires, the MFQ and Internalized Stigma of Mental Illness Inventory—9‐item Version (ISMI‐9*) scales, semi‐structured interviews were then scheduled and conducted with participants by Katie Prizeman between February 1st, 2024, and March 1st, 2024.

We recorded the scale mean (*M*) scores for *N* = 35 subjects as MFQ: *M* = 39.97 and ISMI‐9*: *M* = 2.36. *N *= 18 (51.43%) participants had been diagnosed with clinical depression by a medical professional. *N *= 14 (40%) participants were currently taking psychiatric medication. *N *= 12 (34.28%) of those clinically diagnosed were on medication. There were no other inclusion or exclusion criteria. See Table 2 for all participant characteristics.

### 3.2 Ethical considerations

This study was conducted in accordance with the Declaration of Helsinki. The study proposal was approved by the University Research Ethics Committee (2023‐206‐NW) of the University of Reading on December 12th, 2023. Information about the study was given to the participants, including instructions on the nature of the study, their right to decline to answer any questions they wish, their right to withdraw, and data handling.

Each volunteer who met the inclusion criteria gave written, informed consent. Participants received a debriefing form upon completion of the study. They were also given the option of being contacted about further studies, and if requested, their anonymized data would be publicly shared in the University of Reading's data repository with other researchers.

## 4 DATA COLLECTION

Participants completed demographic questions about age, gender, education, and ethnicity.

### 4.1 Mood and Feelings Questionnaire

We used the MFQ to prescreen participants for depressive symptoms prior to their participation in the study, with higher scores indicating greater depression symptoms. The MFQ (Costello & Angold, 1988) is a 33‐item questionnaire assessing depressive symptoms over the past 2 weeks, with participant responses indicating thoughts, emotions, and behaviors rated on a three‐point scale (0 = *not true*, 1 = *sometimes true*, and 2 = *true*) and is based on DSM‐III‐R symptoms and recommended by the National Institute for Health and Clinical Excellence (2005) for depression screening in children and young people (Daviss et al., 2006; Kent et al., 1997; Rhew et al., 2010; Thabrew et al., 2018; Wood et al., 1995). Total scores, which range from 0 to 66, were determined by the sum of all items.

In order to distinguish clinical from non‐clinical populations, a score of 27 or higher is considered to be the optimal cut‐off point, or best diagnostic confidence, as found by the intersection point of sensitivity (0.78 [95% confidence interval [CI], 0.67–0.89]) and specificity (0.78 [95% CI, 0.66–0.89]; Wood et al., 1995). This score also indicates clinically serious depression (Wood et al., 1995). Example items include, “I felt miserable or unhappy,” “I didn't enjoy anything at all,” “I thought that life wasn't worth living,” and “I found it hard to think properly or concentrate.” This questionnaire is widely used to score depression in young people (Wood et al., 1995).

### 4.2 Internalized Stigma of Mental Illness Inventory—9‐item Version

Participants completed the ISMI‐9* scale. The ISMI‐9* is a nine‐item self‐report questionnaire that yields a score of 1 (*minimal to no internalized stigma*) to 4 (*severe internalized stigma*; Boyd et al., 2014). We determined the total scores by calculating the mean of all items, which range from 1 to 4. The ISMI‐9* also showed strong internal consistency (*α* = 0.86; van Beukering et al., 2022). Example items include, “Stereotypes about the mentally ill apply to me,” “People without mental illness could not possibly understand me,” and “I can't contribute anything to society because I have a mental illness.”

### 4.3 Semi‐structured interviews

A semi‐structured interview method was selected to give participants a voice and an opportunity for exploration. This method enabled participants to direct the interview's course while maintaining flexibility to allow for follow‐up on noteworthy account details that might come up throughout the interviews. Semi‐structured interviews with *N *= 35 young people were scheduled and conducted with participants by Katie Prizeman between February 1st, 2024, and March 1st, 2024. All interviews took place online (using the Microsoft Teams platform). Interviews were conducted until data saturation was reached, meaning that no new information was observed or collected (Guest et al., 2020). Katie Prizeman audio‐recorded, verbatim transcribed, checked for accuracy, and thematically coded the interview transcripts. All interviews took place in English and lasted an average of 65 minutes, with each one lasting between 55 and 75 minutes.

Table 1 presents the qualitative interview guide. Follow‐up prompts included: “Could you elaborate on this?” “Could you provide an example of this or any personal experiences that you might have?” was also used.

**TABLE 1:** Qualitative interview guide.

Focus area	Sample questions and probes from the interview protocol
**Socio‐demographic questions**:	1. Age: 2. Country: 3. Education level (school, university): 4. Ethnicity: 5. Gender (male, female, other, prefer not to say): 6. Have you been diagnosed with clinical depression by a medical professional? 7. Are you on any psychiatric medication?
**Stigma experience and decision to disclose mental health**:	1. Has stigma affected your relationships with others? Can you please explain your answer? 2. Do you think stigma has a large impact on you disclosing or keeping your mental health condition a secret? Please explain and give reasons for your answer. 3. Does talking or not talking about your mental health condition have an impact on your feelings of loneliness and social isolation? Please explain your answer (i.e., what are the impacts of not disclosing feelings of loneliness, isolation, and social withdrawal?). 4. Do you find being alone makes you feel better or worse? Please explain why. 5. What is your preferred mode of sharing your mental health condition (i.e., online platforms, anonymously, communities, etc.)? Please explain your answer. Do you find this helps with feelings of loneliness and social isolation? 6. In your opinion, is socializing better than spending time alone? Please explain and give reasons for your answer.
**Participants recommendations for young people experiencing depression stigma based on personal experiences**:	*Based on your experiences*: 1. What advice can you give young people also living with a mental health condition? How do you think it is best to deal with stigma? Or how would you help others experiencing mental health stigma? 2. What advice would you have for young people for reaching out/talking to others about their mental health condition? 3. How would you explain what the role of talking to other people has in this? Is it helpful and so forth? 4. What are some of the challenges and warnings you could give young people about reaching out? 5. Is there a preferred mode of talking/reaching out you could advise? Please explain your answer. 6. Do you have any tips as to who would be the best sources of support/best young people experiencing stigma to open up to about one's mental health condition and where to find these sources? 7. What advice would you give young people experiencing stigma when making decisions to socialize? 8. Do you have advice for young people experiencing stigma wanting to spend time alone instead of with others and visa versus?

## 5 DATA ANALYSIS

We used NVivo software (version 14) for qualitative data analysis to examine anonymized transcripts. The first author (Katie Prizeman) conducted the analysis utilizing a blend of deductive and inductive methods (Braun & Clarke, 2006; Brink et al., 2006; Fereday & Muir‐Cochrane, 2006). Using the inductive approach, the main researcher (Katie Prizeman) independently reviewed each transcript before extracting and coding important data units using the open‐coding approach (Sarker et al., 2000). Next, using thematic analysis (TA), the research team (Katie Prizeman, Ciara McCabe, and Netta Weinstein) looked for and examined meaningful patterns in the dataset (TA). This method was selected because it is suitable for examining how a group interprets a specific phenomenon (Byrne, 2022; Joffe, 2011).

We developed higher‐order concepts and themes from initial codes by utilizing their shared qualities to produce a codebook. The main researcher (Katie Prizeman) compared all the acquired data, giving considerable thought to and evaluating the primary themes that surfaced during the deductive process. The codebook was continuously improved until data saturation was achieved. At that point, no further themes could be created using the data (Guest et al., 2020). When investigating young people's mental health, the researchers (Katie Prizeman, Ciara McCabe, and Netta Weinstein) accounted for their own sources of bias and preconceived notions, including awareness of depression and mental health stigma (Katie Prizeman, Ciara McCabe, and Netta Weinstein).

## 6 RESULTS

Participants' demographics and clinical characteristics are presented in Table 2.

**TABLE 2:** Participant demographics and clinical characteristics (*N *= 35).

Participant	Age^a^	Gender	Ethnicity	Country	Education level	ISMI‐9* scores (/4)^b^	MFQ scores (/66)^c^	Clinical depression diagnosis?	Taking psychiatric medication?
**P01**	24	Female	African American	Lesotho	University	2	35	Yes	No
**P02**	19	Female	White	UK	University	3	41	Yes	Yes
**P03**	20	Female	Asian	UK	University	2.44	28	No	Yes
**P04**	23	Female	White	UK	High school	2.78	45	Yes	Yes
**P05**	25	Female	White	UK	University	2.22	27	Yes	Yes
**P06**	20	Male	White	South Africa	University	1.67	59	Yes	Yes
**P07**	23	Female	African American	South Africa	University	2.78	38	Yes	Yes
**P08**	19	Female	White	UK	University	2.22	39	Yes	No
**P09**	24	Male	White	Slovakia	High school	2.56	48	Yes	Yes
**P10**	19	Male	White	UK	University	1.33	32	No	No
**P11**	20	Male	White	Germany	University	1.89	50	Yes	No
**P12**	22	Male	Asian	South Africa	University	3.11	57	Yes	Yes
**P13**	20	Female	White	UK	University	1.78	32	No	No
**P14**	19	Female	Asian	UK	University	3.11	42	No	No
**P15**	19	Male	White	UK	University	1.67	28	No	No
**P16**	19	Female	Asian	UK	University	1.67	30	No	No
**P17**	19	Female	White	South Africa	University	2.44	48	Yes	No
**P18**	18	Female	White	UK	University	2.78	58	Yes	Yes
**P19**	19	Male	White	Sweden	University	2.56	56	Yes	Yes
**P20**	18	Female	Asian	UK	University	2.89	38	No	No
**P21**	20	Female	White	UK	University	3.11	33	No	No
**P22**	23	Female	African American	UK	University	2.22	29	Yes	Yes
**P23**	19	Male	White	UK	University	2.22	31	No	No
**P24**	18	Female	White	UK	University	2.56	28	Yes	Yes
**P25**	19	Female	White	UK	University	1.56	38	No	No
**P26**	19	Male	White	UK	University	2.78	28	No	Yes
**P27**	18	Female	Asian	Malaysia	University	2.44	59	No	No
**P28**	21	Male	Asian	Malaysia	University	2.78	32	Yes	No
**P29**	20	Female	Asian	UK	University	3	42	No	No
**P30**	21	Female	Asian	Malaysia	University	2.33	46	No	No
**P31**	19	Male	Other racial‐ethnic group	UK	University	2	28	No	No
**P32**	19	Female	Arabic	UK	University	2.11	35	No	No
**P33**	19	Female	Asian	UK	University	2.33	50	No	No
**P34**	20	Male	African American	South Africa	University	2.22	52	Yes	No
**P35**	19	Male	White	South Africa	University	2.11	37	Yes	Yes

Abbreviations: ISMI‐9*, Internalized Scale Mental Illness Inventory—9‐item Version; MFQ, Mood and Feeling Questionnaire.

*MFQ*—higher scores indicate more depression. All participants completed the long version of the MFQ.

ISMI‐9*—higher scores indicate more internalized stigma (rounded to two decimal places).

^a^ Age at interview.

^b^ ISMI‐9* total score at screening (scores range from 1 to 4). Total ISMI‐9* score is determined by the mean of all items.

^c^ MFQ total score at screening (scores range from 0 to 66). Total MFQ score is determined by the sum of all items.

### 6.1 RQ #1

RQ #1 What are the views on stigma among young people with depression? And how do they see stigma as related to feelings of loneliness, social isolation, withdrawal, and disclosing depression to others?

The majority of participants expressed stigma as a primary challenge when making the decision to disclose their depression. Recurrently, participants voiced depression's disclosure decisions as an important and consequential reaction to previous subjective experiences and views of stigma. Most reported that they withheld more information about their depression as they experienced more stigma, either public or internalized. Many participants recognized that while non‐disclosure may have an initial protective function (i.e., less added stigma, feelings of acceptance by others, and feelings of normality), it also had a long‐term detrimental impact (i.e., increased feelings of loneliness, social isolation, withdrawal, trust issues, and low self‐esteem). They alluded to a vicious loop. For instance, individuals perceived internalized stigma as a direct result of public stigma, and non‐disclosure increased with feelings of loneliness and isolation, which they suggested led them to withdraw. Therefore, directly influencing their relationships and social interactions. Participants also suggested that their internalized stigma experiences increased with public stigma.

Participants also explained the benefits of disclosure. For instance, for many, it led to feeling accepted and secure and receiving understanding and support from close others, disclosure fosters freedom, improves well‐being, lessens feelings of loneliness, isolation, and the need to socially withdraw, as well as providing a sense of empowerment as a way to break the stigma.

These findings are consistent with and confirm our previous research, with participants having voiced both positives and negatives for both disclosure and non‐disclosure of depression, and its direct impact on feelings of loneliness, social isolation, and withdrawal (Prizeman et al., 2024). Similar to our past research, this new sample of participants also described that the solution and most valuable effect is for one to be selectively open about their conditions (i.e., know how much to disclose and how to explain depression during disclosures) (Prizeman et al., 2024).

### 6.2 RQ #2

RQ #2 What recommendations do young people with depressive symptoms have for reaching out to others? What strategies do young people with depression have for others to disclose their mental health and overcome stigma?

Three interrelated broad themes emerged from the data. Themes were used to explore RQ #2, which aimed to create and explore recommendations on how to identify supportive others to disclose depression and overcome stigma in young people with MDD symptoms.

Table 3 presents an overview of the themes and sub‐themes. The quotations below are taken from the original text and have been minimally corrected to ensure proper English use.

**TABLE 3:** Table of themes and sub‐themes.

Themes	Sub‐themes
1. **Social affirmation: identify allies and build meaningful connections**	Understand the purpose of talking and the challenges, warnings, and barriers that come with it Find supportive and trustworthy others and distance from non‐supporting others In‐person mode of talking is preferable and recommended
2. **Self‐affirmation: build a constructive relationship with the self**	Alone time is recommended, necessary, and positive if used productively Find ideal balance between alone and social time Be kind and patient with the self
3. **Societal affirmation:structural changes are needed**	Public health interventions and mental health literacy Health care policies and practices

### 6.3 Theme 1—Social affirmation: identify allies and build meaningful connections

This theme that emerged is directly related to RQ #2 as it explores depressed young people's recommendations on how to strategically and intentionally identify supportive others, build meaningful connections/reach out to others in similar situations, and overcome stigma. The majority of participants expressed stigma as a primary reason for the importance of finding supportive and trustworthy others to reach out to and build meaningful connections with. Participant interviews repeatedly showed the desire to connect with people who share similar experiences, backgrounds, upbringings, and opinions around mental health. Many also voiced that, when opting to disclose, an in‐person conversation would be preferable and most helpful. That said, participants showed an understanding of the challenges and barriers (e.g., others lack of support and understanding, little or no advice, negative responses and reactions, etc.) associated with disclosure to forewarn others before reaching out. Despite this, many encouraged reaching out and explained its importance and purpose.

#### 6.3.1 Sub‐theme a: Understand the purpose of talking and the challenges, warnings, and barriers that come with it

It was important for participants that others recognize the purpose of talking about one's depression. Nearly all participants revealed the role of talking as being very helpful despite stigmatizing responses or adverse past experiences. Participants explained that the purpose of talking is to find relatability, express emotions, create a sense of normalcy, and feel more comfortable when doing so. Additionally, talking serves as motivation for self‐improvement (e.g., talking is good practice), self‐advocacy, hearing other people's perspectives and suggestions for improvement, and creating awareness and better understanding for others.

*“I'd say talking is very helpful like, it's not always going to go perfectly, and you won't get the relief you may be looking for from talking to someone, but like trying to advocate for yourself is like amazing practice. And it will add to the same skill no matter what response you get I think, even though obviously having a supportive response is the best outcome.” (P04, 23, Female)*

*“Sometimes you have to look past the stigma. I feel like you can't take everything that the person says as judging you all too hard because they're trying to find the solution for you. But you might not think that's the best solution for yourself when, actually, it's an entirely different perspective. Maybe something you had never thought about. That can actually be quite helpful.” (P13, 20, Female)*

*“Talking will definitely help you know it will help with all the negative feelings. You know, they might not completely go away, but at least you have, you know, people to share it with every time you feel these feelings and that moment you, you will feel better.” (P14, 19, Female)*

*“Like it's good to practice. Thinking about what's going on for you internally and communicating that to someone else and just advocating for yourself like. Um, a really important skill to have.” (P15, 19, Male)*

*“Talking can help you understand not just yourself, but other people struggling with mental illnesses better. Like helping that person understand what it's like for someone else to have a mental illness and how that they could have like, approach it and just improving their understanding. And that means that person can go and maybe have less of a stigma toward people with mental illnesses and know how to kind of approach and know and kind of understand what people are going through.” (P18, 18, Female)*

*“I think don't expect other people to completely solve your problems. That's not why you're reaching out. You're not reaching out for someone to make everything better. You're reaching out for you.” (21, 20, Female)*

*“I think you just get a sense of normalcy. The more you talk about the issues, the less they seem like such big issues and such like horrible things. I feel like the more you have those open dialogues and talk to people about it, the less, the easier it starts to become. It is very helpful in the sense that you're able to get things off your chest and at the same time it normalizes things.” (P23, 19, Male)*

That said, participants were aware of the challenges, warnings, and barriers that come with disclosing depression. These challenges included not receiving the desired response, others' lack of understanding, unkindness, and dismissiveness, a lack of practical advice, and trust issues. Based on their lived experiences, participants were able to warn others in similar situations about what to expect before reaching out.

*“Like professionals for example will, like, they might say stuff that would hurt you, and things you don't want to hear, but in reality, it's kind of what's necessary. It's what they see and what's dug under that you haven't realized yet. So, you should just kind of keep an open mind, even though it will be hard and can be hurtful, it may be important for you to hear.” (P09, 24, Male)*

*“I would say like you need to realize that no one's going to 100% understand you because they're not living your life in your body. But you need to know that they are there to listen, and if there are professional, they are also there to help you work through your through your thoughts.” (P14, 19, Female)*

*“And I think also just be cautious of like who it is you're talking to and how often you talk to them, because I think sometimes, and I've been there before like it's been me and another friend who I thought I really trusted, where I had talked to a lot about my mental health, and found out she hadn't exactly kept it to herself even though she knew I was telling her certain things in confidence…the next day I find out everyone else in our friend group knows and I wouldn't have necessarily shared these kinds of things with everyone. So, just being mindful of people who you can really trust I think is quite important.” (P21, 20, Female)*

#### 6.3.2 Sub‐theme b: Find supportive and trustworthy others and distance from non‐supporting others

Most participants spoke about social support from others (i.e., partners, family, and friends) as vital to their emotional, mental, and physical health.

*“I would say get help if you can get some support, especially if you have people around you who you trust. Social support is very helpful in helping cope and deal with mental health actually.” (P01, 24, Female)*

*“I felt a lot more comfortable in my skin despite having received a formal diagnosis because I found people that could, you know, support and love me for who I am despite what may happen in my brain.” (P12, 22, Male)*

For various reasons (i.e., broken trust or trust issues, public and internalized stigmatizing attitudes and beliefs, feelings of abnormality and indifference, and generational gap and/or misunderstandings), many expressed difficulties finding, forming, and/or maintaining supportive and trustworthy bonds with others. As a result, participants recommended that others distance themselves from those they feel are untrustworthy, unsupportive, and show a lack of empathy and care.

*“If you feel they are not supportive, and if it's people like family that you cannot completely avoid, maybe limit the kind of mental health conversations you have with them, because they'll end up hurting you at the end of the day and that will just make everything worse, also the stigma.” (P01, 24, Female)*

*“So, it was quite difficult to find someone to relate to or find someone to talk to or be taken seriously without feeling judged or like I am abnormal. But I would recommend, and like I think the older I have got, the more people I've found that I could relate to and trust or had, like, shared upbringings with and those who I don't, I just distance myself.” (P03, 20, Female)*

*“And I am just being weak and insignificant, he makes me feel like I am pathetic, like I am a burden of a child. And that I should just suck it up because I am just being lazy. Like, our generation does that. And I can really see how the stigma affects my relationship with my dad to the point where it is best that we keep our distance. Anyone in similar situations or with toxic relationships, I would encourage to distance themselves.” (P05, 25, Female)*

*“I would say like going back like I don't think I would recommend or open up to my parents as much as I would my friends because obviously, I feel like you're in different stages of your life. I feel like friends appreciate and support more where my mum for example wouldn't understand so would judge a bit more…I mean, because I know I would be judged, my mental health would go more downhill.” (P10, 19, Male)*

Participants also recommended that others in similar situations continue to seek out supportive and trustworthy others, despite adverse past stigma experiences. Participants further voiced that having positive social connections with others leads to stronger social bonds, increased self‐esteem, feelings of fulfillment, growth, acceptance, and reassurance, as well as lessening feelings of shame, loneliness, and social isolation.

*“Like finding people that are there for you, that really love and understand you…that you connect with people that give you the space to be vulnerable, people that give you the space to grow is one of the most beautiful things you can find, and one of the most important social factors that can help us cope better.” (P01, 24, Female)*

*“I recommend and myself choose to disclose to a very specific group of people who are very supportive and even if they don't have the right things to say, just knowing that they have listened to me and given me that space to talk and be vulnerable without worrying about trust issue is enough, yeah. It is very reassuring that it's um, it puts an ease, sort of relief on me that I have that support.” (P02, 19, Female)*

*“I mean, when I talk to people about my mental health struggles and being hospitalized because my self‐harm got to a point of being so bad, it does make me feel less lonely because, like, somebody out there does understand me, people do know about it and will still make you feel worthy and supported, and want to know me as a person. There are people out there, you just need to find them.” (P05, 25, Female)*

*“Finding and having supportive people around you, I feel like does make people closer and sort of build stronger relationships.” (P35, 19, Male)*

#### 6.3.3 Sub‐theme c: In‐person mode of talking is preferable and recommended

Regardless of their experiences with stigma, many participants choose to engage in face‐to‐face mental health conversations and recommend others to do the same. They expressed the in‐person mode of talking as preferable for several reasons, including physical contact or interactions (i.e., being able to receive a hug), seeing facial expressions and body reactions, feelings of a safe space or supportive environment, more genuine and heartfelt conversations, and lessening feelings of loneliness and isolation.

*“I think that people will actually realize that it is an experience, like this is my life, rather than it's just words on a screen, that kind of thing.” (P02, 19, Female)*

*“I feel like it's more about the connection you make with the person, so you could actually tell if they are, you know, really listening…you could tell by their body language and eye contact. But in‐person is also nice because you know they are there; they want to be. It feels like you're in a safe space. That is usually the best way.” (P06, 20, Male)*

*“I get quite anxious sometimes about speaking online or on the phone cause like it kind of limits the interactions you can have with people, like body language and the way in which they respond, which are so powerful, and just texting, yeah, you never know if the person on the other end is actually listening. And I think in‐person, it's just the safest option…I would say if others are able to speak about their mental health and do have a group of people who they can trust in a supportive environment, um, like I said, like just having that other person there with you, it just makes you feel safer.” (P07, 23, Female)*

*“I much prefer talking to someone in‐person. Yeah, because in‐person can feel a bit more heartfelt and genuine, like their responses and their support. And you can have a hug and stuff like that. And because it's social interaction, like in‐person, it also helps a lot, like taking away the loneliness and isolation. So, it's a lot better.” (P08, 19, Female)*

*“Yes, it has helped. Uh, just because there is someone who actually wants to listen, and it has helped with feeling lonely and, you know, isolating myself for long periods. Something so small, like just being able to receive that hug after a difficult chat, can make such a difference.” (P11, 20, Male)*

Despite the benefits of in‐person conversations, participants understood that such conversations require a certain amount of openness, trust, and comfort and will be more difficult for some than others. Participants further acknowledged that stigma is a major factor in why having in‐person conversations regarding one's mental health can be more difficult. For example, stigma can make people feel judged, weak, and insignificant due to their condition. Still, many suggest that in‐person conversations are preferable and of the greatest benefit, while others start by sharing online or anonymously as a way to work up to in‐person conversations.

*“Yeah, I think definitely in person is one way because you can actually read the other person's body language. They can read your body language and you can understand the intent of the conversation. But if someone is not quite there yet and like unable to open up in person, I think anonymously is a good start to getting there in a way because you're getting the most objective, umm, perspectives from other people, which I find very helpful. But yeah, I would say definitely go for in‐person as a first choice if you are able to or at least have this as the end goal in mind if you're not there yet.” (P05, 25, Female)*

*“I feel like because you have that comfort of the person being there face‐to‐face and so on, and it also feels like you're with them physically, that is very helpful. So, you know they're there for you. And I feel like if people don't like talking that much or are not at the point of talking to others face‐to‐face because there is always that fear of being judged, you know, do the online thing where you just type to somebody. I feel like typing is a lot easier than talking to start off.” (P13, 20, Female)*

*“I feel like if they're too scared of being judged and treated in a certain way, then yes, I would suggest anonymously and then they can work up to opening up in‐person about who they actually are kind of thing. It's kind of like stages you have to go through to actually be more comfortable with it.” (P19, 19, Male)*

*“No one wants to open up to get further stigmatized, so yeah, I do get it. Like, if talking in‐person causes stress and makes you feel weak and unworthy, what's the point, you know? Like, obviously, no one wants to feel that way. Essentially, there needs to be that level of trust. Rather, start online or something and work your way up until you have that supportive environment.” (P34, 20, Male)*

### 6.4 Theme 2—Self‐affirmation: build a constructive relationship with the self

This theme involves depressed young people's recommendations on how to build a relationship and be kind and patient with themselves, even despite past and present stigma experiences. Many recommend and explain alone time as both necessary and positive if used productively. Despite the potential benefits of solitude time, participants repeatedly highlighted stigma's impact on young people's feelings of loneliness, social isolation, withdrawal, and self‐esteem from lengthy periods of aloneness. They encourage others to find a balance between alone time and social time. Reasons for finding an ideal balance and the benefits of balance are based on participant interviews and are discussed below.

#### 6.4.1 Sub‐theme a: Alone time is recommended, necessary, and positive if used productively

The majority of participants recommended and expressed the necessity of alone time. Recurring reasons for alone time included valuable time to build and establish a good relationship with the self (i.e., lean about oneself, find comfort in being alone), for hobbies and relaxation, time used to regain strength, reflect (i.e., events of the day, journaling), and recharge one's social battery, and to avoid existing and/or added stigmas. Furthermore, many said feelings of loneliness and isolation were minimized when time alone was spent in a positive manner.

*“I do find that I regain my strength just by being on my own, because I just resented me when I when I go for, like let's say I go for holiday or there's a period where I have to interact with a lot of people and I'm with people all the time, that can be very draining, even if it's just a small amount of people, even if it's the people I love. And I think being myself, yeah, I do regain my strength and sort of have that time to just by being myself and recharge my social battery. So usually, it is very good for me to just be on my own. It feels very necessary.” (P05, 25, Female)*

*“I think it's great to have a relationship with yourself first and be able to depend on yourself and not always be dependent on being with other people because people will always disappoint and judge you. This way, I get to avoid the judgments and work on having a relationship with me first.” (P07, 23, Female)*

*“Like sometimes when you're like, so overwhelmed by people and like you don't wanna deal with, like, the stigma anything more, like being alone definitely does bring a sort of comforting feeling and that does help.” (P16, 19, Female)*

*“Since I'm journaling and all that, I don't feel lonely. I'm not isolating for the wrong reasons, I just really enjoy my alone time. But I only socially isolate and withdraw for very long periods when there is something unhealthy on my mind and nothing I do is enjoyable and that can be and create a very lonely place.” (P20, 18, Female)*

Despite potential benefits of time alone, it did not come naturally to all. Participants repeatedly articulated the importance of having self‐awareness of one's mental health struggles and how one is feeling for alone time to be healthy and positive.

*“OK, this is what I personally do when I spend when alone. If I'm feeling a certain way, I tried to sort of have a discussion with myself and understand why I feel this way and then if 10 minutes pass by and I'm still thinking about the same thing then I know it's time to move on to something else like I literally then try to distract myself. Also thinking I lot doesn't help, but at the same time you can't ignore it and it is if it's not there. So that's why I'm like I have a time limit because it keeps me self‐aware. If I'm still thinking about the same thing. Then I'm like, no. Now it's too much. The alone time is not healthy.” (P07, 23, Female)*

*“Like it can be positive to be alone, but it's also very easy to sort of spiral into a thing of bad thoughts if you're not using it purposefully…But when it gets to the point where I am literally in bed just watching the clock that's when I know, OK, I need to at least go outside for a walk or do something meaningful.” (P22, 23, Female)*

*“So, obviously being if you are at the point where you are self‐aware enough of your own mental health struggles, that you should be able to identify one as pathological or not like, when you are just going deeper into a hole or if being alone is just for you to resent and harm yourself, that's obviously not healthy. But like if its productive and like feels useful or you are able to learn about yourself or just do something you enjoy even if it's just taking a bath, then it can be really good for your mental health.” (P29, 20, Female)*

*“So, like for me, I know that it's fine for myself to be alone when I actually get out of bed, take a shower, make my bed and all that. And I read a book or I do something, but I know that it's not good if I am, like, it's 3:00 PM and I'm stuck in bed or something like that. So, it's just about identifying your own behaviors. And it's just about getting to the point where you're self‐aware enough to know what is good and what's not. But yeah, spending time in your own is good if you can see the signs of when it's good or bad.” (P31, 19, Male)*

#### 6.4.2 Sub‐theme b: Find ideal balance between alone and social time

Despite alone time being necessary and positive if used appropriately, participants expressed that too much alone time is disruptive to well‐being and felt to be unhealthy.

*“And also, be aware of how much time you're spending alone, because that can also become a habit…Also try, like it's not a bad thing to socialize, but also try to have time for yourself because again, you can't if you're always socializing, it's like you're running away from what's going on internally. But also, you need to have that time and focus on being with other people…I feel like it's a good thing too because also you have to remember that people would always be there for you.” (P07, 23, Female)*

*“Socializing always can get very overwhelming, but also being alone can make you feel very, very lonely and you like, need a good mix of both for it to be healthy.” (P16, 19, Female)*

*“If you know the time spent alone gets out of control and if it's really harmful and like you can't get yourself out of that withdrawal, then I think getting professional help would be the best.” (P24, 18, Female)*

Interviewees often expressed that social and alone time are equally important, and participants recommended that finding a healthy balance between the two is essential.

*“It's important to connect with each other, to feel connected with other people. I mean they all like equally important… I think we need to like, connect to others as like the world but also need to reflect and yeah digest things that have happened. So, yeah it's sort of a balance of both.” (P04, 23, Female)*

*“Yeah, although I think alone time is better, there should be a balance of both, like it depends on the sort of person you are. Like sometimes everyone like, needs a little like, alone time where you're doing your own thing, you know, but that social interaction with others and like, connecting with the world around you and that is important. Yeah, so not too much of anything.” (P14, 19, Female)*

*“Personally, I find that I prefer being alone. But I think when you're alone, it's hard to distract yourself from necessarily the thoughts you're having or how you're feeling. But also, I think it is incredibly important to be able to support yourself, because at the end of day, not everyone is always gonna be there for you. So maybe at the start of your journey with mental health, look for other people for that support system. So, you know you're not alone, but when you do feel comfortable enough that also exploring how you can help yourself and support yourself is also vital, cause you need to be able to self‐soothe. It's really about having a good balance of both.” (P19, 19, Male)*

Participants further described the importance of feeling comfortable, unpressured, and included when choosing to socialize. Many expressed social exclusion and stigma as the main causes of feelings of loneliness, isolation, added stigma, and ultimately social withdrawal. Participants' experiences of added stigma, which directly link to feelings of discomfort, prejudice, and pressure in social settings, explain a key conclusion of this research. This made it more difficult for one to socialize and connect with the world around them, form and maintain social connections, and create the desired balance between social time and alone time.

*“…socializing is something that I've struggled with because of the stigma for sure. And then you just you slowly learn, what are the best ways to approach people and how you feel most comfortable. But yeah, completely. I do recommend it for sure. Just don't put too much pressure on yourself if you're not feeling comfortable, so that's what I'd say. Just be yourself too.” (P17, 19, Female)*

*“I'm trying to find ways that I do feel comfortable around people because I do feel better at times when I'm with other people, I do want to socialize. I just don't want to be judged, so I definitely think it's a combination of both. But when you are truly struggling, reaching out to other people and being around other people who make you feel assured is your best bet.” (P19, 19, Male)*

*“Yeah, based on my experiences I think that feeling included does lessen that lonely feeling for me. And would lead to more socialization naturally. And if somebody feels excluded and stigmatized for the label that they've got, and then there's that feeling of being judged, you do feel like an outcast and it feels like being socially excluded. And that makes it very easy to withdraw and bring back the lonely feelings.” (P25, 19, Female)*

*“You need to be able to be alone and be comfortable with being alone. But you also need to be able to socialize and be comfortable with being around others.” (P26, 19, Male)*

*“If they're not sure, I think comfortability. If they're comfortable enough, I think, I think socializing has important benefits to us, so I would suggest that they socialize. But I do know that it's not always easy and but it's always expected of us. I don't think you should do something that you feel like is an expectation.” (P31, 19, Male)*

#### 6.4.3 Sub‐theme c: Be kind and patient with the self

Based on past experiences, participants recommended that when experiencing stigma, young people remind themselves that it is due to others lack of understanding and knowledge of mental disorders. Many frequently expressed the importance of being kind and patient with oneself and giving oneself more grace. Kindness, patience, and graciousness were recommended as core components for self‐healing.

*“And not to be too hard on yourself to try and be more patient with yourself. Just because you have a mental illness, doesn't you know, mean that you're a bad person.” (P01, 24, Female)*

*“Yeah, it's helpful to know that someone's going through the same thing, and that there isn't anything wrong with me because of my diagnosis. I don't need to punish myself for it because my depression isn't me, it is just something I am working through…Yeah, I would remind others to just remember that and be kind to themselves.” (P02, 19, Female)*

*“And then I sort of just need to remind myself, no, I do have a vital health issue and it is valid, a lot of people struggle with it.” (P05, 25, Female)*

*“And I think like just be kind to yourself. Like, it's not just like almost seeing it as a part of you, not the whole of you. Like your mental health is, just because you've been told that you have XY, or Z doesn't mean that that's what you are. That's just a small part of what makes up the whole of you. Just remember that and to just be kind.” (P22, 23, Female)*

*“So, it's just, it's just about giving yourself grace and taking it day by day and it's just about being understanding of your own mental health because at the end of the day other people are always going to have those judgments…” (P27, 18, Female)*

Many participants further expressed that it was important to attend to and accept one's experiences and that listening to the body was a way of healing. Acceptance of emotions played a particularly prominent role:

*“Yeah, because I sometimes also, even when I'm feeling depressed, I just let myself feel that I just allow myself. I don't want to force myself to go out and I don't know, take over the world when I'm feeling down. I would say it's really important to let yourself feel what you are feeling, otherwise, the emotions just to build up later on. So, just allow yourself to feel the feeling so that it can pass, and you can go on, yeah.” (P05, 25, Female)*

*“And I always, I always think you're just allowing yourself to feel your feelings because I know, like, a lot of the times when you're going through something, somebody would be like, ‘You shouldn't think about it or try not to think about it. Put your effort into something else. Then you don't have to think about it.’ I don't believe in that. I think you should think about the things that are upsetting you. I think you should talk about what's upsetting you, and I think you should feel upset. And I think just because if you, if you like, just experience the intensity and feel it up until its peak, that's when you actually heal instead of just like mildly feeling the pain or being mildly upset. And then it just lingering. So rather just feel it and feel it to its full extent and then letting it go on its own.” (P05, 25, Female)*

*“But I find that being, or just working with the mental health issue, rather than working against it in a way is healing. So, if you feel like I wanna stay in bed all day sometimes, just do it. Just allow yourself to feel that and to experience that work from there. So weirdly like just intuitive, because sometimes your body and your mind just need whatever you are needing to do, even if it seems like it's pathological.” (P28, 21, Male)*

*“…your feelings are valid, and you are allowed to feel hurt or. Like you're allowed to feel suppressed, but just let your thoughts out. Even if you're feeling bad, feeling your emotions and having those reactions are positive and it just it just feels like a relief when you can actually let it out.” (P33, 19, Female)*

### 6.5 Theme 3—Societal affirmation: structural changes are needed

The theme focused on the recommendations made by depressed young people for future structural interventions aimed at preventing stigma. In response to the stigmas that affect mental health, participants repeatedly highlighted the need to do more to inform others, develop interventions, and develop policies on mental health‐related stigmas. The research and interventions that follow are based on participant interviews and draw inspiration from recommendations made by young people.

#### 6.5.1 Sub‐theme a: Public health interventions and mental health literacy

The majority of participants spoke of stigma as a core reason for others’ lack of understanding and knowledge related to mental illnesses. Participants suggested that to increase mental health literacy, public health policies, programs, services, and interventions be put in place to influence mental health stigmas and shape societal beliefs and norms. Many explained that the primary goal of these interventions is to create awareness, improve mental health knowledge, and lessen societal preconceptions about those with mental illnesses. For example, mental health promotion programs may unintentionally shape society's ideas by increasing awareness, educating, and perpetuating responsibility for those who engage in these stigmatizing attitudes, beliefs, and behaviors.

*“Yeah, so having more posters, signposting, and like help centers or care programs and numbers that you can call 24/7 to help, and just creating that overall awareness, I do think is important to get over or at least lessen the stigma.” (P02, 19, Female)*

*“I guess it's just about educating people about it from a really young age, like and sort of a like stopping those negative perceptions of it before they kind of get ingrained in someone. So, like having mental health programs or classes about it with children at school so that like future generations, hopefully they'll just grow up, being less stigmatizing toward it.” (P22, 23, Female)*

*“Governments should get more involved for sure…more posters or signposting around where people can speak to different communities and get access to help, and just aiming to promote well‐being and create that awareness of mental health stigmas in the community, things like that is quite important.” (P30, 21, Female)*

*“I think just educating society and letting them know that certain things that used to happen in the past don't happen now…I think one problem that the older generation have is they're not able to adapt to understanding how mental health and stigma is changing. So, I think educating people on all levels is quite important to improve prejudgments.” (P32, 19, Female)*

#### 6.5.2 Sub‐theme b: Health care policies and practices

Participant interviews repeatedly recommended having better health care policies and practices as a way to improve and overcome stigmas, such as the accessibility and availability of government mental health care services. Health care policies and practices mentioned by interviewees included improving the accessibility of mental health services (i.e., access and availability of counselors, for instance, in schools, universities, and hospitals), outreach programs (i.e., mobile health visits, home calls, and peer support groups), and greater access to mental health clinicians who support patients in understanding health care systems and adhering to treatment programs. Such advice is illustrated by the following participant quotations:

*“And I think probably providing more counseling services and free public help services especially because a lot of people can't afford to pay for the help…things like that would help.” (P02, 19, Female)*

*“So, for me, my mental health can be quite debilitating to the point where you know, like I can't leave the house, I can't do basic things…having to walk from the sofa to the fridge is a struggle. And like having public health services that could perhaps do home calls or something like that I think could reach a lot of people also struggling could be something that becomes quite popular.” (P04, 23, Female)*

*“…when I look back to when I was hospitalized, and I was fortunate enough to be in a private clinic, and what I find really helpful to this day was the medical team assigned to me guiding me through my treatment plan and making sure I also understand it outside of here…but I know not many people have that opportunity to be in private clinics so just making sure the same standard if I can say is also given in public clinics…having a team that is able to make sure you are 100% understanding of what they are doing and what you need to continue to do after.” (P05, 25, Female)*

*“I mean, obviously if they have access to like whether it's a so like psychological professional or GP or that sort of thing, I think, yeah, definitely go get all the actual resources you can, also if you can afford it…I know from experience even once you open yourself up to getting professional help, actually managing to get an appointment or find someone who is available is not easy. Like, in my school, for example, we had one mental health counselor that was always booked out. So, maybe government focus on having more of those resources and have them easier to access so when you do need them, they are there, you know.” (P23, 19, Male)*

## 7 DISCUSSION

We elicited the views of young people with depression symptoms on stigma and explored how it has affected their loneliness, social isolation, withdrawal, and disclosing depression to others. We also obtained the recommendations young people with depressive symptoms have for social, personal, and societal changes to counter stigma and support mental health.

We found stigma to be the main challenge for young people deciding to disclose their depression. Young people discussed the protective function of non‐disclosure (i.e., less added stigma, feelings of acceptance and normality) and its long‐term detrimental impacts (i.e., increased stigma, loneliness, social isolation, withdrawal, as well as trust and self‐esteem issues; Bos et al., 2009; De Hooge et al., 2010; Link et al., 2001; Mayer et al., 2022; Prizeman et al., 2024). They also discussed the benefits that come from deciding to disclose one's depression. Disclosure benefits included lower feelings of loneliness, social isolation, and the need for social withdrawal; increased feelings of acceptance, inclusion, support, and understanding from close others; and improved well‐being (Frattaroli, 2006; Garcia & Crocker, 2008; Prizeman et al., 2024, 2023). Overall and similar to previous work (Bos et al., 2009; Corrigan et al., 2018; Prizeman et al., 2024; Rüsch et al., 2017), we found young people with depression symptoms suggested selective disclosure to be the solution and of most value.

We identified three main themes on social, self‐, and societal affirmation in participants’ interviews, specifically in relation to RQ #2, that is, recommendations young people with depressive symptoms have for reaching out to others and the strategies they have for others to disclose their mental health and overcome stigma.

*Social affirmation: identify allies and build meaningful connections* focused on the helpfulness of talking despite stigmatizing responses and past experiences. Talking has an important role in that it is suggestive of improved well‐being. Specifically, our findings imply that talking about one's depression can create a sense of normalcy and comfort. Research has consistently shown positive links between disclosing mental disorders, quality of life, and recovery, with those choosing to disclose having received support, acceptance, and non‐stigmatizing responses (Bril‐Barniv et al., 2017; Reavley et al., 2018; Rüsch et al., 2019). Our findings support past research indicating that disclosure increases comfort and potentially improves well‐being (Corrigan et al., 2010; Rüsch et al., 2014). Similar to past research, our findings further highlighted the importance of finding trustworthy and supportive others for continued willingness to disclose and the recommendation to distance oneself from unsupportive, untrustworthy others (Añez et al., 2005; Bos et al., 2009; Cervantes & Castro, 1985; Chronister et al., 2013; Derlega et al., 2004; Keefe et al., 1979). Throughout our interviews, young people showed to be very aware of the challenges and warnings that come with talking about one's depression (i.e., stigmatizing responses and lack of others understanding, trust, and dismissiveness; Bos et al., 2009; Valle & Levy, 2009), and warned those in similar circumstances about what to expect before reaching out. With little research existing on the topic, many participants from our findings reported in‐person conversation to be their preferred mode of talking and recommended this to others as well (Bulkes et al., 2022). While acknowledging that in‐person conversations can be difficult for some, it allowed individuals to feel a sense of physical comfort, provide genuine and appropriate responses based on others' facial expressions and body language, and lessen feelings of loneliness and isolation.

*Self‐affirmation: build a constructive relationship with the self*—Despite past and present stigma experiences, participants talked about the importance and necessity of alone time and being able to find an ideal balance between alone and social time. Alone time seemed to have surprisingly more positive than negative outcomes on young people's experiences. Specifically, our findings showed that alone time is deemed invaluable for building a constructive relationship with oneself—in essence, a platform for one to establish a sense of confidence, build on one's self‐identity, and find comfort in being alone despite their depression. Research has consistently shown that for young people with mental disorders, alone time can be a purposeful way to reach important identity‐formation developmental stages (Goossens, 2013; Thomas & Azmitia, 2019). Many benefits exist and include better adjustment, increased emotional regulation, increased ability to self‐reflect, and better enjoyment of solo leisure activities (Goossens & Marcoen, 1999; Larson, 1997; Long & Averill, 2003). In line with our findings, and in addition to being necessary and recommended, when alone time is positive and productive, opportunities to spend time alone increase and have the ability to lessen feelings of loneliness and isolation (Borg & Willoughby, 2022; Corsano et al., 2019).

In our sample, most participants also expressed the value of social time and the importance of finding an ideal balance between being alone and social. Therefore, socializing allows individuals to connect with the world around them and form and maintain healthy social connections. Consistent with past research, our results have suggested that the desire to socialize increases when individuals feel comfortable, destigmatized, and unpressured in social situations, which in turn lessens feelings of loneliness, isolation, and social withdrawal (Bargh et al., 1996; Prizeman et al., 2023; Sayce, 1998).

Overall, findings within this theme highlighted the importance of self‐compassion and that being kind, patient, and gracious with oneself are core aspects of the recovery process. Extensive research demonstrates the link between higher self‐compassion and lower symptoms of clinical depression and anxiety (Bluth et al., 2016, 2015; Marsh et al., 2018; Muris & Otgaar, 2020; Neff, 2003, 2020). Therefore, it is an active ingredient in prevention and psychological interventions for young people with mental disorders (Arimitsu, 2016; Foxx et al., 2020; Trust, 2021). Further, while our findings with young people highlighted the importance of self‐compassion and its benefits (i.e., increased self‐acceptance, self‐confidence, decreased depression and anxiety symptoms, etc.), future research can aim to explore questions of how and for whom self‐compassion interventions work and improve understanding of the reasons for the associations between self‐compassion and anxiety and depression in young people.

*Societal affirmation: structural changes are needed*—Participants discussed strategies for the development of public health interventions, mental health literacy, and health care policies in response to the stigmas associated with mental disorders. Our results showed that others’ lack of knowledge and understanding was a recurring topic in interviews. Participants advise solutions focused on creating awareness and improving knowledge to reduce societal preconceptions about mental illnesses, which may be utilized to frame future policy conversations regarding population‐level stigma reduction strategies. Specific to our results, examples of health care policies and practices to reduce stigma based on young people's recommendations are in line with past studies and include: accessibility and availability of governmental health care services (i.e., access and availability to counselors and mental health care workers); outreach programs (i.e., mobile home visits and peer support groups); and improved access to policies that help individuals navigate health systems and adhere to treatment plans (i.e., clinician support for patients long‐term adherence and care plans; Katz et al., 2011; Schabert et al., 2013; Woodgate et al., 2017). Nevertheless, our findings add to previous research that suggests many unknown structural underpinnings of stigma that function in a variety of social and health contexts (Bolster‐Foucault et al., 2021).

### 7.1 Strengths, limitations, and future directions

An important strength of our study is the use of in‐depth, semi‐structured interviews. Unlike quantitative research, our study design gives the opportunity for a thorough investigation of strategies for young people with depression symptoms to disclose mental health and overcome stigma based on lived experiences. We recruited 35 participants from various contexts (mental health care, general community, schools, and universities), resulting in a sizable sample of volunteers from various ages, and education levels, which is another strength of our study for qualitative research standards. In addition, we included young people, both with and without a medical professional's clinical depression diagnosis. See Table 2.

A limitation of this study is the relatively large number of female participants (*N* = 22; 62.86%). Another limitation is the limited information that was gained on participant backgrounds and countries impeding a thorough analysis of the influence these elements have on offering suggestions to others in similar situations.

Future studies might concentrate on involving more individuals from a wider range of geographic areas. Therefore, it is important to rigorously examine the extent to which recommended strategies for others to disclose mental health and overcome stigma are representative of the larger population of young people exhibiting depression symptoms. Future studies may also investigate the impact of an individual's sociocultural conditions on their stigma experiences and outcomes.

## 8 CONCLUSIONS

This study explored the experiences of young people with depression and their suggestions for mitigating mental health stigma by fostering a healthy self‐relationship, fostering supportive social connections, and utilizing supportive societal resources. While participants acknowledged that non‐disclosure of depression has an early protective role, they also felt that non‐disclosure had long‐term detrimental impacts, creating a vicious cycle (i.e., stigma leads to loneliness, isolation, and withdrawal, which impacts relationship experiences and social interactions). Nevertheless, there were other advantages to disclosure, including empowerment to end the stigma, feeling supported, encouraging independence, and enhancing well‐being. Our research indicates that social, self‐, and societal affirmation are important topics for decreased stigma, improved well‐being, and recovery for people struggling with disclosure decisions and depression stigmas.

These strategies highlight the need for guided policies and programs that provide mental health support to young people and public awareness campaigns that guide young people to appropriate resources (i.e., support and intervention) via governmental public health bodies to directly reduce depression stigma.

## AUTHOR CONTRIBUTIONS

**Katie Prizeman**: Conceptualization; investigation; writing—original draft; methodology; writing—review and editing; visualization; formal analysis; project administration; software; data curation; resources; validation. **Netta Weinstein**: Conceptualization; visualization; validation; writing—review and editing; methodology; supervision; project administration. **Ciara McCabe**: Conceptualization; methodology; validation; visualization; writing—review and editing; project administration; supervision.

## CONFLICT OF INTEREST STATEMENT

The authors declare no conflicts of interest.

### PEER REVIEW

The peer review history for this article is available at https://publons.com/publon/10.1002/brb3.70028.

## Data Availability

Anonymized and deidentified data are publicly available and can be accessed through the University of Reading's Research Data Archive. Prizeman, Katie (2024): Data supporting: “Strategies to overcome mental health stigma: Insights and recommendations from young people with major depressive disorder (MDD).” University of Reading. Dataset. https://doi.org/10.17864/1947.001319
